# Autoantigen cross-reactive environmental antigen can trigger multiple sclerosis-like disease

**DOI:** 10.1186/s12974-015-0313-9

**Published:** 2015-05-13

**Authors:** Catherine J Reynolds, Malcolm J W Sim, Kathryn J Quigley, Daniel M Altmann, Rosemary J Boyton

**Affiliations:** Lung Immunology Group, Section of Infectious Diseases and Immunity, Division of Infectious Diseases, Department of Medicine, Hammersmith Hospital, Imperial College London, Room 8N22, Commonwealth Building, Du Cane Road, London, W12 ONN UK; Section of Molecular Immunology, Division of Immunology and Inflammation, Department of Medicine, Hammersmith Hospital, Imperial College London, Du Cane Road, London, W12 ONN UK

**Keywords:** Multiple sclerosis, CD4 T cell, T cell receptor transgenic, Epitope, Cross-reactivity, Autoimmunity, TCR, Exposome

## Abstract

**Background:**

Multiple sclerosis is generally considered an autoimmune disease resulting from interaction between predisposing genes and environmental factors, together allowing immunological self-tolerance to be compromised. The precise nature of the environmental inputs has been elusive, infectious agents having received considerable attention. A recent study generated an algorithm predicting naturally occurring T cell receptor (TCR) ligands from the proteome database. Taking the example of a multiple sclerosis patient-derived anti-myelin TCR, the study identified a number of stimulatory, cross-reactive peptide sequences from environmental and human antigens. Having previously generated a spontaneous multiple sclerosis (MS) model through expression of this TCR, we asked whether any of these could indeed function *in vivo* to trigger CNS disease by cross-reactive activation.

**Findings:**

A number of myelin epitope cross-reactive epitopes could stimulate T cell immunity in this MS anti-myelin TCR transgenic model. Two of the most stimulatory of these ‘environmental’ epitopes, from *Dictyostyelium* slime mold and from *Emiliania huxleyi*, were tested for the ability to induce MS-like disease in the transgenics. We found that immunization with cross-reactive peptide from *Dictyostyelium* slime mold (but not from *E. huxleyi*) induces severe disease.

**Conclusions:**

These specific environmental epitopes are unlikely to be common triggers of MS, but this study suggests that our search for the cross-reactivity triggers of autoimmune activation leading to MS should encompass epitopes not just from the ‘infectome’ but also from the full environmental ‘exposome.’

## Findings

For multiple sclerosis (MS), as with other autoimmune diseases, susceptibility depends to some extent on a number of predisposing genes, disease being conferred to a greater degree by uncharted environmental risk factors [1]. The impact of environmental risk factors is inferred, for example, from many studies showing that migration as a child from a country of low prevalence to a country of high prevalence imposes the risk of the new country of domicile [2]. Epidemiological studies aimed at characterizing the environmental determinants have been challenging. Those that have received the most attention are UV-exposure and infectious history [3]. Of the many infectious agents that have been considered, Epstein-Barr virus (EBV) is arguably the pathogen for which there is most supporting evidence, including evidence from the relationship between severe infectious mononucleosis and MS [4]. The case for a microbial pathogen in etiology has been argued in relation either to cross-reactive stimulation of autoreactive T cell receptors by related pathogen sequences (molecular mimicry) or to pathogen-driven CNS inflammation and bystander activation [5,6].

A recent study used deep-sequencing data and proteomic databases to generate an algorithm for naturally occurring T cell receptor (TCR) ligands [7]. This encompassed a search for naturally occurring, cross-reactive, environmental ligands for a prototypic, multiple sclerosis patient-derived T cell receptor specific for myelin basic protein epitope. An implication was that in autoimmune etiology, we may now need to consider not just pathogen exposure but all of the structurally related potentially cross-reactive protein-derived ligands to which we may be exposed in our environment - the exposome in its broadest sense [8].

## Hypothesis

Having previously reported modeling of MS immunology and pathology in an HLA-DR15 and TCR transgenic model utilizing the same myelin basic protein (MBP) specific TCR as used by Birnbaum *et al*. [9], we, here, took the opportunity to investigate whether ‘environmental’ peptides implicated by the structural algorithm could indeed act by cross-reactivity to induce disease.

In the model that we have previously reported, expression of the Ob1.A12 TCR on a high proportion of murine CD4 and CD8 cells results in spontaneous, chronic demyelinating disease, highly reminiscent of human MS [9,10]. This strain, termed Line 7, shows disease that is initially mediated by migration to the CNS of IFNγ-secreting and then IL-17-secreting cells and encompasses intermolecular epitope spread from the initial MBP 85-99 epitope. While spontaneous disease can develop at any stage from around 16 weeks to 1 year or older, synchronous disease can be induced in young-adult mice by the injection of MBP peptide in adjuvant.

## Methods

Line 7 mice have been previously described by us and carry HLA-DR15 with an anti-MBP (85-99)-specific TCR (clone Ob1.A12) on an Aβ^o^ background [9,10]. Mice were maintained in individually ventilated cages and were used in experiments as age- and sex-matched young adults. Mouse experiments were performed within UK Home Office legislation under the terms of the Project License PPL 70/8110 granted for this work under the ‘Animals (Scientific Procedures) Act 1986’. Local ethical review and formal approval had also been obtained through the Imperial College Ethical Review Process Committee.

Unprimed splenocytes from previously described Line 7 HLA-DR15 and TCR transgenics carrying an anti-MBP (85-99)-specific TCR (clone Ob1.A12) on an HLA-DR1501/Aβ^o^ background (*n* = 6) were cultured in triplicate on pre-coated IFNγ ELISpot plates with 0.25, 2.5, or 25 μg/ml of each of the 12 test peptides, MPB 85-99, and a negative control peptide (*Burkholderia pseudomallei* AhpC, BPSL2096 51-70 KDFTFVCPTEIVEFAKLAKQ which stimulates potent CD4 T cell responses in HLA-DR1501/Aβ^o^ transgenic mice [11]). Cells were cultured for 72 h. Data are expressed as SFC/10^6^ splenocytes and shown as mean values ± SEM.

Line 7 mice were used to test the encephalitogenic capacity of test peptides. Young male mice received 300 μg peptide subcutaneously in the flank using CFA supplemented with *Mycobacterium butyricum* at day 0 and 50 μg peptide subcutaneously in CFA at day 6. Pertussis toxin at a dose of 200 ng intraperitoneally was administered on days 0, 2, 6, and 8. Mice were scored daily for signs of neurological disease using an established disease score scale according to the following criteria: 0, normal; 1, limp tail; 2, impaired righting reflex or waddling gait; 3, partial hind limb paralysis; 4, total hind limb paralysis; 5, total limb paralysis.

## Results

**Figure 1:**
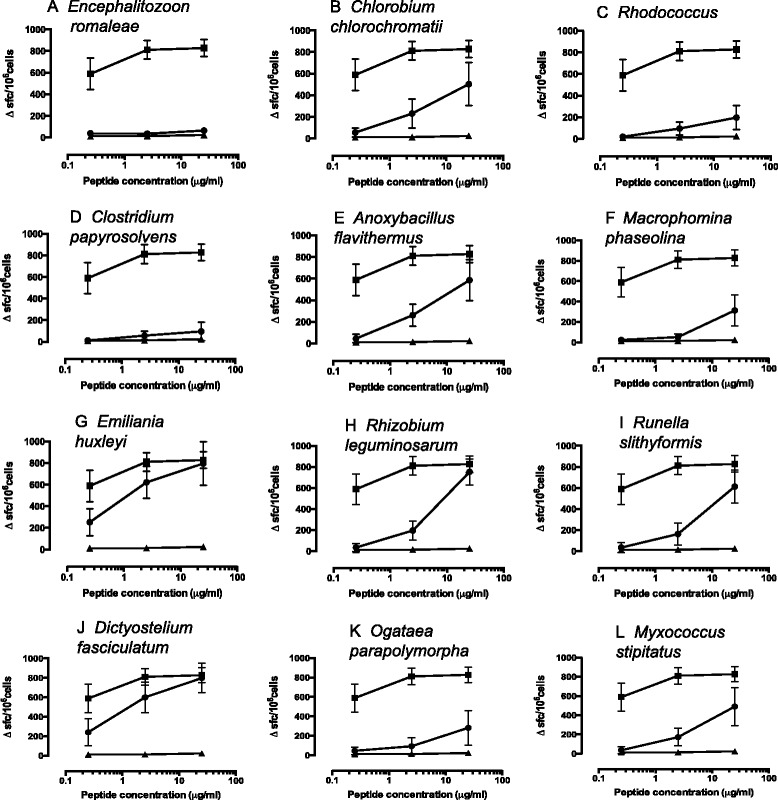
T cell assays show the relative functional avidity of environmental ligands. **(A-L)** Unprimed splenocytes from previously described HLA-DR15 and TCR transgenics carrying an anti-MBP (85-99) specific TCR (clone Ob1.A12) on an HLA-DR1501/Aβ^o^ background (*n* = 6) were cultured in triplicate on precoated IFNγ ELISpot plates with 0.25, 2.5, or 25 μg/ml of each of the 12 test peptides, MPB 85-99, and a negative control peptide (HLA-DR1501 binding peptide from *Burkholderia pseudomallei*, AhpC, BPSL2096 (51-70) KDFTFVCPTEIVEFAKLAKQ which stimulates potent CD4 T cell responses in HLA-DR1501/Aβ^o^ transgenic mice [11]). Cells were cultured for 72 h before plate development. Data are expressed as SFC/10^6^ splenocytes and shown as mean values ± SEM. In each case, MBP 85-99 positive control peptide is indicated as closed squares, test peptide as closed circles, and negative control peptide as closed triangles. Test peptide identities were as follows: (A) *Encephalitozoon romaleae*, FGVKIHFFKQRNSL; (B) *Chlorobium chlorochromatii* CaD3, VFGNVHFFKNTGSA; (C) *Rhodococcus* sp. AW25MO9, AAQRIHFFKNLSLL; (D) *Clostridium papyrosolvens*, LNKNIHFFKNLPLP; (E) *Anoxybacillus flavithermus*, RLSVVHFLRANAVS; (F) *Macrophomina phaseolina* MS6, AAQNVHFWKALNQL; (G) *Emiliania huxleyi* CCMP1516, STARVHFWRSRSSE; (H) *Rhizobium leguminosarum*, DVSKVHFFKGNGQT; (I) *Runella slithyformis* DSM 19594, HRAKLHFFKDENLK; (J) *Dictyostelium fasciculatum*, YKHKIHFFKNEVLE; (K) *Ogataea parapolymorpha* DL-1, IEAAIHFYKGLAVY; (L) *Myxococcus stipitatus* DSM14675, SSARLHFFRALPHP.

We started by reappraising in functional T cell assays the relative functional avidity of the environmental ligands described by Birnbaum *et al*. [7]. These encompassed peptide sequences from *Encephalitozoon romaleae*, *Chlorobium chlorochromatii*, *Rhodococcus* sp. AW25MO9, *Clostridium papyrosolvens*, *Anoxybacillus flavithermus*, *Macrophomina phaseolina*, *Emiliania huxleyi*, *Rhizobium leguminosarum*, *Runella slithyformis*, *Dictyostelium fasciculatum*, *Ogataea parapolymorpha*, and *Myxococcus stipitatus* (Table 1). Screening Line 7 splenocytes, IFNγ responses from unprimed mice, we observed a spectrum of response to the epitopes that had been previously identified as cross-reactive for this receptor (Figure 1). Two epitopes, from *E. romaleae* and from *C. papyrosolvens* elicited virtually no T cell response. Most peptides yielded a response that was significant but with reduced potency of at least 100-fold relative to the wild-type MBP 85-99 peptide. Two of the peptides, from hypothetical proteins of *D. fasciculatum* and *E. huxleyi*, induced responses within 1-log of the MBP epitope. *D. fasciculatum* is a cellular slime mold while *E. huxleyi* is a photosynthetic ocean plankton. It is uncertain why some of those epitopes predicted to be stimulatory did not elicit a response in our line 7 transgenics. However, even among lines expressing the same human TCR pair, there will be differences in response profile, determined for example by founder-specific differences in TCR transgene expression.

**Table 1:** **Peptide sequence and origin**

**Peptide sequence** ^**a**^	**Mr**	**#**	**Species**	**Protein of origin**
**ENPVVHFFKNIVTP**	**1641**	**MBP**	**Homo sapiens**	**Myelin basic protein (85-99)**
FGVKI**HFFK**QRNSL	1721	A	*Encephalitozoon romaleae*	UDP-N-Acetylglucosamine pyrophosphorylase
VFGN**VHFFK**NTGSA	1525	B	*Chlorobium chlorochromate CaD3*	Hypothetical protein
AAQRI**HFFK**NLSLL	1658	C	*Rhodococcus sp. AW25m09*	Hypothetical protein
L**N**KNI**HFFKN**LPL**P**	1695	D	*Clostridium papyrosolvens*	Exonuclease ABC C subunit domain protein
RLS**VVHF**LRANAVS	1569	E	*Anoxybacillus flavithermus*	Spore germination protein
AAQN**VHF**W**K**ALNQL	1640	F	*Macrophomina phaseolina*	Hypothetical protein MPH
STAR**VHF**WRSRSSE	1706	G	*Emiliania huxleyi*	Hypothetical protein
DVSK**VHFFK**GNGQT	1564	H	*Rhizobium leguminosarum*	ABC transporter
HRAKL**HFFK**DENLK	1783	I	*Runella slithyformis*	Aldo/keto reductase
YKHKI**HFFKN**E**V**LE	1832	J	*Dictyostelium fasciculatum*	Hypothetical protein DFA
IEAAI**HF**Y**K**GLAVY	1595	K	*Ogataea parapolymorpha*	Component of TOM complex
SSARL**HFF**RALPH**P**	1636	L	*Myxococcus stipitatus*	Hypothetical protein
KDFTFVCPTEIVEFAKLAKQ	2315	Bp	*Burkholderia pseudomallei*	AhpC (51-70)

^a^Bold type face indicates sequence homology with MBP^85-99^.^.^Data in this table are based on information within reference 7.

**Figure 2:**
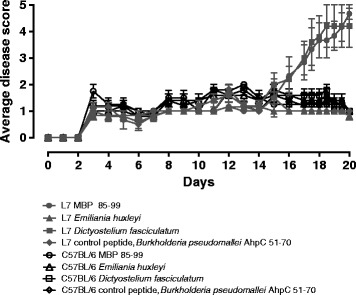
Induction of autoimmune paralysis in TCR transgenic multiple sclerosis model by a cross-reactive *Dictyostelium* slime mold peptide. Previously described HLA-DR15 and TCR transgenics carrying an anti-MBP (85-99) specific TCR (clone Ob1.A12) and HLA-DR1501, A^o^ were used to test the encephalitogenic capacity of test peptides. Young male mice received 300 μg peptide subcutaneously in supplemented CFA at day 0 and 50 μg peptide subcutaneously in CFA at day 6. Pertussis toxin at a dose of 200 ng intraperitoneally was administered on days 0, 2, 6, and 8. Mice were scored daily for signs of neurological disease using an established disease score scale according to the following criteria: 0, normal; 1, limp tail; 2, impaired righting reflex or waddling gait; 3, partial hind limb paralysis; 4, total hind limb paralysis; 5, total limb paralysis. HLA-DR15 and TCR transgenics carrying the anti-MBP (85-99)-specific TCR (clone Ob1.A12) and HLA-DR1501, A^o^ were administered MBP 85-99 peptide (closed circles, *n* = 6), *Dictyostelium fasciculatum* YKHKIHFFKNEVLE peptide (closed squares, *n* = 6), *Emiliania huxleyi* CCMP1516 STARVHFWRSRSSE peptide (closed triangles, *n* = 6), control peptide, *Burkholderia pseudomallei* AhpC (51-70) KDFTFVCPTEIVEFAKLAKQ (closed diamonds, *n* = 6), C57BL/6 mice administered MBP 85-99 peptide (open circles, *n* = 5), *Emiliania huxleyi* CCMP1516 STARVHFWRSRSSE peptide (open triangles, *n* = 5), *Dictyostelium fasciculatum* YKHKIHFFKNEVLE peptide (open squares, *n* = 5), and *Burkholderia pseudomallei* AhpC (51-70) KDFTFVCPTEIVEFAKLAKQ (open diamonds, *n* = 5). MBP, myelin basic protein.

Of the peptides that had induced cross-reactive T cell responses in the line 7 mice, we tested the two most stimulatory, from *D. fasciculatum* and from *E. huxleyi*, for the ability to induce disease. These were compared to MBP 85-99 peptide as a positive control and to an HLA-DR15-binding CD4 T cell epitope from *Burkholderia* as a negative control [11]. The *Dictyostyelium* slime mold epitope could indeed induce severe disease (Figure 2). Paralysis was of a time-course and severity similar to that induced by MBP 85-99. Postmortem neuropathology was not undertaken in this study, but our previous published work has correlated the neurological disease score used here with neuropathology (9,10). The ability to induce disease using environmental antigen-derived sequences was not a simple correlate of functional avidity, since the similarly stimulatory sequence from *E. huxleyi* did not induce disease. Further work will be required to dissect the underlying mechanism for this difference, which may relate to divergence in the cytokine profiles elicited.

## Interpretation

To be clear, we interpret this as proof of principle rather than specific evidence that exposure to slime mold sequences are pathogenic in clinical MS: the Ob1.A12 TCR stimulated by this peptide is not a public receptor across MS patients in general, and therefore, one would not generalize a case based on its specific cross-reactivities. Sequencing of the TCR repertoire from MS patients has demonstrated substantial diversity in myelin epitope-specific disease-implicated receptors [12,13]. Clearly, a further caveat in considering the implications of environmental cross-reactivities is that of predicted epitopes; not all could actually stimulate T cells in our model, and not all peptides that could stimulate could induce disease. Notwithstanding these caveats and following on from the cross-reactivities modeled by Birnbaum and colleagues and our subsequent demonstration that at least one example from an antigen in the wider environment can trigger MS-like disease, we can consider a new chapter of autoimmunity research, analyzing such triggers and their contribution to disease. This newly places the experimental analysis of the relationship between an individual’s exposome, their immune repertoire, and their susceptibility to autoimmunity within the domain of structural and functional immunology [14].

It has long been a given in MS research that there are environmental risk factors relating to geography and, in particular, latitude [http://www.msif.org/wp-content/uploads/2014/09/Atlas-of-MS.pdf]. The implication of the model described here is that it will now be important to consider in this context not just old favorite candidate environmental influences such as vitamin D exposure but also local flora and fauna in its widest sense.

## Abbreviations

CFA: Complete Freund’s Adjuvant
CNS: central nervous system
EBV: Epstein-Barr virus
HLA: human leukocyte antigen
IFNg: interferon g
MBP: myelin basic protein
MS: multiple sclerosis
SD: standard deviation
TCR: T cell receptor
